# Adverse Events of Saffron (*Crocus sativus* L.): Systematic Review of Current Evidence

**DOI:** 10.1002/hsr2.72212

**Published:** 2026-04-27

**Authors:** Fatemeh Sadat Hasheminasab, Mahdie Hajimonfarednejad, Seyede Maryam Najibi, Mohammad Hashem Hashempur

**Affiliations:** ^1^ Pharmacology Research Center Zahedan University of Medical Sciences Zahedan Iran; ^2^ Department of Genetic, School of Medicine Zahedan University of Medical Sciences Zahedan Iran; ^3^ Research Center for Traditional Medicine and History of Medicine, Department of Persian Medicine, School of Medicine Shiraz University of Medical Sciences Shiraz Iran; ^4^ Canadian College of Integrative Medicine (CCIM) Montreal Quebec Canada; ^5^ Department of Health Services Management, School of Health Management and Information Sciences Shiraz University of Medical Sciences Shiraz Iran

**Keywords:** adverse effect, adverse event, complication, *Crocus sativus*, saffron, systematic review, traditional Persian medicine

## Abstract

**Background:**

*Crocus sativus* L., commonly known as saffron, is a widely used spice with a rich history of culinary and medicinal applications. This systematic review aims to compile human data from studies on monopreparations of *C. sativus*, including stigma powder and other extracts, to evaluate their safety.

**Methods:**

Databases, including the Web of Science, Scopus, and PubMed Central, were searched up to August 2024 using the keywords “saffron” or “*Crocus sativus*” for clinical trials, case reports, and case series.

**Results:**

A total of 102 clinical trials and a single case report were included in this review. Nearly 78% of the trials evaluating the monopreparation of *C. sativus* addressed safety concerns and reported the complications that occurred. Approximately 56% indicated that one or more adverse effects were experienced during the intervention period. The estimated incidence of adverse events related to *C. sativus* consumption is around 17.5%. The most frequently reported side effects were gastrointestinal problems, which were minor and self‐limiting in most cases.

**Conclusion:**

The findings suggest that *C. sativus* is generally well tolerated. However, due to some potentially significant adverse events associated with its medicinal use at higher doses or prolonged administration, clinical monitoring should be considered.

## 1 Introduction

Saffron (*Crocus sativus* L.) is a sophisticated spice with culinary and therapeutic importance [1]. It expands from bulbs and is part of the family Iridaceae and the genus *Crocus*, native to regions such as Spain, India, and Iran [1, 2]. *C. sativus* has its roots over thousands of years in traditional Chinese medicine, exploiting its health benefits like antimicrobial, antispasmodic, and anti‐inflammatory ones [2, 3].

The elite Persian medical scholars from the medieval period regarded *C. sativus* due to its potent antidepressant, anxiolytic, analgesic, and aphrodisiac properties [4, 5, 6]. This plant constitutes a set of leaves, a bulb, and a flower that contains stamens, tepals, and stigmas, while the spice itself comes specifically from the dried stigmas [7]. Extensive research has proven the same results which focus on *C. sativus*, confirming its antidepressant, antioxidant, antitumor, antimicrobial, antifungal, antihypertensive, antidiabetic, immunomodulatory, gastroprotective, and neuroprotective properties [1, 4, 7, 8, 9].

The chemical evaluation of the stigmas of *C. sativus* has shown about 150 constituents, both volatile and nonvolatile. Nonetheless, less than half of those compounds have been identified so far [10]. The three most important biologically active compounds are as follows: (1) Crocin, a carotenoid pigment responsible for the yellow–orange color of the spice; (2) Safranal, a volatile compound responsible for the peculiar odor of *C. sativus*; and (3) Picrocrocin, which gives the characteristic taste and bitterness of *C. sativus* [11, 12].

According to recent research, over 80% of the world's population has made use of some form of herbal therapy, with the numbers being different based on ethnicity, country, gender, age, and even particular ailments [13, 14, 15, 16]. Difficulties in accessing healthcare, their high costs, personal preferences, and the perceived safety of medicinal herbs have contributed to the increased usage of herbalism [15, 17, 18]. Given their popularity, it is crucial to have up‐to‐date risk and benefit assessments on these herbal products [17]. Herbal medicines are often regarded by the public as completely safe, but there have been serious reported side effects involving some of these herbal treatments, like liver failure, cancer, allergic reactions, renal failure, colon rupture, changes in mental state, and even death [15, 19].

Despite the widespread and increasing use of *C. sativus* in various clinical areas, including mood disorders, anxiety, metabolic syndrome, and neurological diseases, a systematic and comprehensive evaluation specifically addressing the safety profile and side effects of this medicinal plant in humans has not been conducted to date. Most of the available reviews have focused on the therapeutic efficacy of *C. sativus*. However, the general belief in the inherent safety of medicinal plants, as well as the increase in their self‐administration or prescription in vulnerable populations (such as patients on polypharmacy), has made the serious gap in knowledge of *C. sativus* immunology an urgent clinical concern. The lack of reliable data on side effects, drug interactions (especially with anticoagulants), and dose–response patterns can jeopardize clinical decision‐making. Therefore, compiling a systematic review of human studies with the aim of summarizing the available evidence on the side effects of *C. sativus* not only fills the gap in the literature but also seems essential for physicians, researchers, and health policymakers to use this widely used medicinal plant safely and rationally. Therefore, this study is the first systematic review to focus exclusively on the adverse effects of *C. sativus* monopreparations in humans. This review aims to integrate all available evidence to provide a comprehensive picture of the safety profile of this widely used herb.

## 2 Materials and Methods

### 2.1 Search Strategy

A comprehensive systematic literature search was conducted using the electronic databases Web of Science, Scopus, and PubMed Central, covering the period from their inception until the end of August 2024. The search employed the terms “saffron” OR “*Crocus sativus*” AND “trial” OR “case report” OR “case series” within the subject, abstract, and keywords. Additionally, the reference lists of all qualifying articles were reviewed to identify other potentially relevant studies.

This review included data from placebo‐controlled clinical trials, case reports, and case series. Only human studies focusing on mono‐preparations of *C. sativus*, such as *C. sativus* stigma powder or its alcoholic, hydroalcoholic, or aqueous extracts, were considered. Studies involving *C. sativus* in combination with other herbs, homeopathic formulations, and those examining pure phytochemical compounds of *C. sativus*, as well as animal and in vitro research, were excluded.

The screening and evaluation process of the studies was carried out independently by two reviewers. For greater transparency, the process was systematically implemented as follows:

a. Initial screening (title and abstract): After the initial search and removal of duplicates, all identified records (title and abstract) were independently reviewed by two reviewers (S. M. N. and M. H.) based on the inclusion and exclusion criteria. In case of disagreement between the two reviewers, the final vote was made by a third reviewer (F. S. H.).
b. Full‐text evaluation: Studies that were initially deemed eligible were then independently evaluated in full text by the two reviewers (S. M. N. and M. H.). Again, in cases of disagreement, the third reviewer (F. S. H.) made the final decision.
c. Data extraction: Relevant data (study characteristics, population, dose, duration of use, side effects, etc.) were extracted independently by two reviewers using a data extraction form. The results extracted by both reviewers were compared, and any discrepancies were resolved by discussion and, where necessary, by the opinion of a third reviewer (F. S. H.).

This procedure (independent data extraction by at least two reviewers and resolution of disagreements by a third reviewer) was performed to reduce bias and increase reliability in the systematic review process. No formal statistical analysis of the primary data was performed. This systematic review was performed following the Preferred Reporting Items for Systematic Reviews and Meta‐analyses (PRISMA) guidelines [20].

### 2.2 Assessing the Quality of Studies

The methodology of this systematic review also included a systematic review of bias in clinical trials, which is crucial for judging the reliability of the collected data. The standard Cochrane's Risk of Bias‐2 (ROB‐2) tool was used to assess the risk of bias in clinical trials. This tool judges the risk of bias in the following five main areas: (1) Bias due to the randomization process, (2) Bias due to deviation from the intended intervention, (3) Bias due to missing outcome data, (4) Bias due to outcome measurement, and (5) Bias due to selection of the reported outcome. Finally, based on the status of these five areas, an overall judgment of risk of bias was made for each study.

The assessment process was as follows: Risk of bias assessment was carried out independently by two assessors (S. M. N. and M. H.). Next, a third evaluator (F. S. H.) reviewed and confirmed the assessments, and resolved any disagreements between the two initial evaluators.

## 3 Result

### 3.1 Clinical Trials

**Figure 1:**
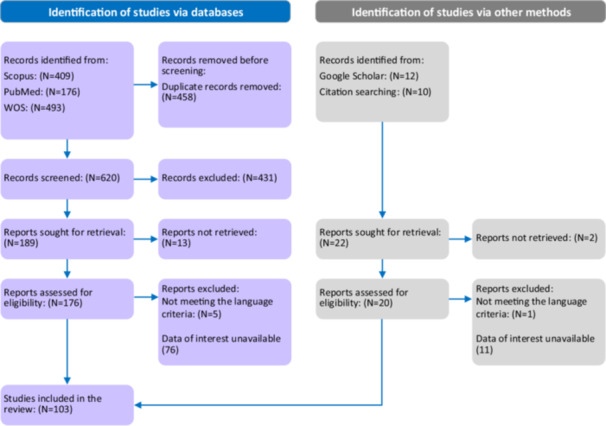
Flow diagram for the study selection process.

A total of 102 clinical trials were included in this review. Of these, 44 studies reported adverse events, 35 indicated the absence of adverse events, and 23 trials did not provide any information regarding side effects. The process of study selection is presented in Figure 1.

#### 3.1.1 Randomized Trials Reporting Adverse Events

In total, 44 studies reported adverse events associated with *C. sativus*. Given the wide range of potential applications for this substance, the study populations in these trials were notably diverse. They included healthy volunteers [21], patients with type 2 diabetes mellitus [22], individuals with rheumatoid arthritis [23], those suffering from irritable bowel syndrome [24], women experiencing premenstrual syndrome [25], patients diagnosed with Alzheimer's disease [26], individuals with age‐related macular degeneration [27], patients with cardiovascular conditions [28], and subjects with attention‐deficit/hyperactivity disorder [29] (see Table 1). The duration of the interventions varied, ranging from three consecutive nights to 12 months.

**Table 1:** Placebo‐controlled trials of saffron preparations reporting adverse events.

	Trial	*N* total/*N* interventional group	Patient population	Preparation, daily dose (dosage form)	Treatment duration	Adverse events in saffron group (*N*)		Adverse events in control group (*N*)	
1	Broadhead et al. [27]	93/93 (cross over)	Adults (> 50 years) with mild/moderate AMD (age‐related macular degeneration) and vision > 20/70 Snellen equivalent in at least 1 eye	*C. sativus*, 20 mg, daily (capsule)	6 months (3 months of either saffron or placebo followed by cross‐over to the other arm of the study)	Death (malignancy) (1) Death (pneumonia) (1) Aortic valve replacement (1) Fall (1) Neovascular AMD (7) SCC requiring excision (1) Idiopathic pancreatitis (1) Bowel cancer (1) (None of the SAEs were thought to be linked to the use of saffron supplementation)			
2	Kazemi et al. [28]	42/21	Cardiovascular patients	*C. sativus* extract (hydroalcoholic extract), 30 mg, daily (pill)	6 months	Digestive problems (3)		—	
3	Lopresti and Smith [30]	62/31	Recreationally active adults	*C. sativus* extract, 28 mg, daily (tablet)	6 weeks	More vivid dreams (2) Increased muscle pain (1) Increased thirst (1)		Increased headaches (1) Sleep disturbances (1)	
4	Kashani et al. [31]	68/34	Female with sexual dysfunction	*C. sativus* extract, 15 mg, twice daily (capsule)	6 weeks	Headache (4) Insomnia (2) Dry mouth (1) Nausea (3) Constipation (1) Swatting (2) Vomiting (1)		Headache (4) Insomnia (2) Dry mouth (1) Nausea (2) Constipation (2) Swatting (2) Vomiting (1)	
5	Blasco‐Fontecilla et al. [32]	63/36	Children and adolescents with attention‐deficit/hyperactivity disorder	*C. sativus* extract (Saffr'Activ), 30 mg, daily (capsule)	6 weeks	Side effects—not detailed (10)		side effects—not detailed (7)	
6	Pazoki et al. [33]	56/28	Adults with attention‐deficit/hyperactivity disorder	*C. sativus*, 30 mg/day (capsule) (Both groups received 30 mg/day methylphenidate hydrochloride)	6 weeks	Insomnia (17) Headache (14) Abdominal pain (13) Decreased appetite (16) Nausea (3) Itching (4) Cough (2)		Insomnia (18) Headache (15) Abdominal pain (11) Decreased appetite (14) Nausea (5) Itching (3) Cough (3)	
7	Pachikian et al. [34]	66/34	Subjects presenting mild to moderate sleep disorder associated with anxiety	*C. sativus* extract, 15.5 mg, daily	6 weeks	Palpitations (1)			
8	Lopresti et al. [35]	120/80	Adults with poor sleep	*C. sativus* extract (affron), 14 mg, or 28 mg, daily (capsule)	28 days	14 mg: Tiredness (1) Headaches/migraine (2) Yellowing of skin (1) Weight gain (1)	28 mg: Headaches/migraine (2) Dry mouth (2) Increased urination (1) Nausea (1) Diarrhea (1) Dizziness (1) Chest pain (1)	Irritability (1) Increased tiredness (1) Headaches/migraine (1) Vivid dreams (1) Dry mouth (1) Diarrhea (1) Bloating (1) Dizziness (1)	
9	Lopresti and Smith [36]	86/43	Women experiencing menopausal complaints	*C. sativus* extract (affron), 14 mg, twice daily (tablet)	12 weeks	Flatulence (3) Nausea/bloating (2) Reflux (1) Migraine/headache (2) Dry mouth (1) Joint pain (1)		Constipation (2) Decreased appetite (1) Body odor (1) Weight gain (1) Pressure in head (1) Nightmares (1) Fatigue (1) Increased hot flushes (1)	
10	Jackson et al. [37]	56/30	Healthy adults	*C. sativus* extract, 30 mg, twice daily (capsule)	8 weeks	Greater number of incidents of gastrointestinal upset			
11	Rajabi et al. [38]	120/40	Women with premenstrual dysphoric disorder	*C. sativus*, 15 mg, twice daily (capsule)	2 weeks	Gastrointestinal symptoms (2) Insomnia (1) Increased menorrhagia (5)		Fluoxetine group: Gastrointestinal symptoms (8) Headache (3) Anxiety (4) Insomnia (3) Increased menorrhagia (3)	Placebo group: Gastrointestinal symptoms (9) Headache (3) Anxiety (1) Increased menorrhagia (2)
12	Pour et al. [39]	76/38	Nonalcoholic fatty liver disease patient	*C. sativus*, 100 mg, daily (tablet)	12 weeks	Allergic reaction (1)			
13	Hamidi et al. [23]	66/33	Patients with active rheumatoid arthritis	*C. sativus*, 100 mg, daily (tablet)	12 weeks	Stomach pain (1)		Stomach pain (1)	
14	Ali‐Akbari‐Sichani et al. [40]	60/30	Primiparous women whose gestational age was 40 weeks or longer	*C. sativus*, 250 mg, daily (capsule)	Three consecutive nights	Weakness (1) Heartbeat and nausea (2)		Increase in fetal movements (1)	
15	Akhondzadeh et al. [41]	73/36	Overweight women with mild to moderate depression	*C. sativus*, 15 mg, twice daily (capsule)	12 weeks	Nightmare and anxiety (1)		Sleep disturbance and increased appetite (1)	
16	Najafabadi et al. [24]	66/33	Patients with irritable bowel syndrome	*C. sativus* extract, 30 mg, daily (capsule)	6 weeks	Headache (3) Dry mouth (3) Nausea (3) Daytime drowsiness (4) Constipation (3) Sweating (2) Vomiting (2)		Headache (4) Dry mouth (4) Nausea (4) Daytime drowsiness (4) Constipation (3) Sweating (3) Vomiting (3)	
17	Broadhead et al. [42]	100/100 (cross over)	Patient with mild/moderate age‐related macular degeneration	*C. sativus*, 20 mg/day, at midday (capsule)	3 months	Death (urinary sepsis) (1) Fall (1) Neovascular AMD (2) SCC requiring excision (1) Cataract progression (1) Idiopathic pancreatitis (1) Bowel cancer (1)		Fall (2) Neovascular AMD (1) Cataract progression (1) Acute torticollis (1) Acute myocardial infarction (1) Hip fracture (1) Bowel cancer (1) Bradycardia requiring Pacemaker insertion (1) Incarcerated hernia (1)	
18	Baziar et al. [29]	54/27	Children with attention‐deficit/hyperactivity disorder	*C. sativus*, 20–30 mg/day (capsule)	6 weeks	Headache (2) Dry mouth (2) Nausea (2) Insomnia (2) Decreased appetite (2) Sweating (2) Vomiting (2)		Headache (5) Dry mouth (3) Nausea (4) Insomnia (5) Decreased appetite (5) Sweating (2) Vomiting (4)	
19	Ahmadpanah et al. 2019 [43]	50/25	Older people with major depressive disorders	*C. sativus* extract, 60 mg/day (capsule)	6 weeks	Tiredness (1)		Headache (3) Vertigo (2) Tiredness (3) Sleep (2)	
20	Lopresti et al. [44]	80/40	Youth aged 12–16 years, with mild‐to‐moderate anxiety or depressive symptoms	*C. sativus* extract, 14 mg, twice daily (tablet)	8 weeks	Headaches (1)		Nausea and stomach pain (1) Headaches (5)	
21	Milajerdi et al. [22]	54/27	Patients with type 2 diabetes mellitus	*C. sativus* extract, 15 mg, twice a day (capsule)	8 weeks	Headache (1)		Headache (1)	
22	Shakiba et al. [45]	54/27	Outpatients with fibromyalgia	*C. sativus* extract, 15 mg/day (capsule)	8 weeks	Abdominal pain (1) Nausea (2) Diarrhea (1)		Nausea (4) Decreased appetite (3) Headache (2)	
23	Asr Badr et al. [46]	48/27	Infertile men with clinical varicocele after varicocelectomy	*C. sativus*, 60 mg, once every other day (capsule)	6 months	Irritative urinary syndrome (1)			
24	Esalatmanesh et al. [47]	50/25	Patients with mild to moderate obsessive‐compulsive disorder	*C. sativus* extract, 30 mg/day, 15 mg twice a day (capsule)	10 weeks	Headache (2) Dry mouth (3) Nausea (3) Daytime drowsiness (2) Constipation (2) Sweating (2) Vomiting (2)		Headache (3) Dry mouth (3) Nausea (3) Daytime drowsiness (2) Constipation (3) Sweating (4) vomiting (3)	
25	Ghajar et al. [48]	66/33	Patients with major depressive disorder accompanied by anxious distress	*C. sativus*, 30 mg/day (capsule)	6 weeks	Headache (2) Nausea/vomiting (2)		Headache (2) Vertigo (5) Nausea/vomiting (2) Drowsiness (2) Gastritis (2) Anger/rage (3) Palpitation (1)	
26	Jafarnia et al. [49]	40/20	Patients with mild to moderate generalized anxiety disorder	*C. sativus*, 450 mg (capsules)	6 weeks	Constipation (1) Polydipsia (1) Headache (2)			
27	Kashani et al. [50]	64/32	Women aged 18–45 years with mild to moderate postpartum depression	*C. sativus* extract, 15 mg, twice daily (capsule)	6 weeks	Headache (1) Dry mouth (2) Nausea (4) Daytime drowsiness (1) Constipation (4) Sweating (1)		Headache (5) Dry mouth (4) Nausea (4) Daytime drowsiness (4) Constipation (4) Sweating (3)	
28	Tabeshpour et al. [51]	60/30	Mothers suffering from mild‐to‐moderate postpartum depressive disorder	*C. sativus*, 15 mg, twice/day (tablet)	8 weeks	Bleeding gums (probably platelet dysfunction) (1) Gastrointestinal disorder (2) Lack of sleep (1) Oversleeping (1) Low breast milk supply (2)			
29	Akhondzadeh et al. [26]	46/23	Patients with mild to moderate Alzheimer's disease	*C. sativus*, 30 mg/day, 15 mg twice per day (capsule)	16 weeks	Dizziness (2) Dry mouth (3) Fatigue (1) Hypomania (2) Nausea (2)		Dizziness (3) Dry mouth (1) Fatigue (2) Nausea (1)	
30	Akhondzadeh et al. [52]	54/27	Patients with Alzheimer's disease	*C. sativus*, 30 mg/day, 15 mg twice per day (capsule)	22 weeks	Vomiting (1) Dizziness (2) Dry mouth (5) Fatigue (1) Hypomania (1) Nausea (2)		Vomiting (7) Dizziness (5) Dry mouth (3) Fatigue (4) Nausea (6)	
31	Gout et al. [53]	60/31	Mildly overweight, healthy women	*C. sativus*, 176.5 mg/day (capsule)	8 weeks	Nausea, diarrhea, and reflux (5)			
32	Mansoori et al. [54]	20/10	Patient with the diagnosis of major depressive disorder	*C. sativus*, 15 mg, twice daily (capsule)	4 weeks	Dry mouth (3) Restlessness (2) Anxiety (2) Constipation (1) Daily drowsiness (1) Morning drowsiness (1)		Dry mouth (2) Tachycardia (1) Constipation (1) Nausea (1) Reflux (1) Abdominal pain (1) Headache (1) Dizziness (1) Daily drowsiness (1)	
33	Safarinejad et al. [55]	260/130	Infertile men with idiopathic oligoasthenoteratozoospermia	*C. sativus*, 60 mg/day (capsule)	26 weeks	Headache (1) Nausea (2) Hypomania (1) increased appetite (1)			
34	Modabbernia et al. [56]	36/15	Married male patients with major depressive disorder	*C. sativus* extract, 15 mg, twice per day (capsule)	4 weeks	Daytime drowsiness (1) Nausea (1) Decreased appetite (3) Dry mouth (3) Nervousness (1) Restlessness (1) Morning drowsiness (1) Increased appetite (1)		Abdominal pain (1) Daytime drowsiness (1) Nausea (3) Decreased appetite (1) Dry mouth (2) Increased appetite (1)	
35	Moosavi et al. [57]	60/30	Patients with moderate depressive disorders	*C. sativus* extract, 40 mg and 80 mg, Twice per day (capsule)	6 weeks	Saffron 40: Nausea or vomiting (1) Dizziness (1) Headache (1) Drowsiness (2) Insomnia (1) Saffron 80: Nausea or vomiting (1) Dizziness (1) Headache (1) Drowsiness (2) Insomnia (2)			
36	Shahmansouri et al. [58]	44/20	Patients who were suffering from depression after performing percutaneous coronary intervention	*C. sativus* extract, 30 mg/day (capsule)	6 weeks	Morning drowsiness (1) Constipation (1) Decreased appetite (4) Dry mouth (1)		Daytime drowsiness (1) Morning drowsiness (1) Constipation (3) Nervousness (1) Decreased appetite (2) Dry mouth (4)	
37	Akhondzadeh et al. [59]	30/15	Patient with mild to moderate depression	*C. sativus* extract, 30 mg/three times per day (capsule)	6 weeks	Anxiety (4) Decreased appetite (2) Increased appetite (1) Nausea (2) Headache (3) Dry mouth (1) Hypomania (2) Constipation (2) Urinary retention (1)		Anxiety (1) Increased appetite (5) Sedation (6) Nausea (1) Headache (2) Dry mouth (7) Hypomania (1) Constipation (5) Urinary retention (5)	
38	Noorbala et al. [60]	40/20	Patient with mild to moderate depression	*C. sativus* extract, 30 mg/day, twice per day (capsule)	6 weeks	Anxiety (3) Decreased appetite (2) Increased appetite (5) Sedation (1) Nausea (2) Headache (3)		Anxiety (6) Decreased appetite (5) Increased appetite (2) Nausea (4) Headache (6) Sexual dysfunction (4) Tremor (4) Sweating (3)	
39	Moshiri et al. [61]	40/20	Patients with mild to moderate depression	*C. sativus* extract, 30 mg/day, twice per day (capsule)	6 weeks	Anxiety (4) Decreased appetite (4) Stomach pain (4) Tremor (3) Nausea (5) Headache (3) Sweating (2) Heart pounding (4)		Anxiety (2) Decreased appetite (2) Stomach pain (2) Tremor (1) Nausea (2) Headache (1) Sweating (1) Heart pounding (2)	
40	Akhondzadeh Basti et al. [62]	40/20	Patient with depression	*C. sativus*, 15 mg, twice per day (capsule)	8 weeks	Anxiety (4) Decreased appetite (5) Increased appetite (1) Sexual dysfunction (3) Tremor (2) Nausea (3) Headache (2) Sweating (2) Heart pounding (3) Insomnia (3)		Anxiety (7) Decreased appetite (4) Increased appetite (3) Sexual dysfunction (5) Tremor (5) Nausea (4) Headache (5) Sweating (3) Heart pounding (2) Insomnia (3)	
41	Agha‐Hosseini et al. [25]	50/25	Women aged 20–45 years with regular menstrual cycles and experience of premenstrual syndrome	*C. sativus* extract, 15 mg twice a day (capsule)	6 months	Decreased appetite (3) Increased appetite (4) Sedation (1) Nausea (2) Headache (3) Hypomania (2)		Decreased appetite (2) Increased appetite (2) Sedation (2) Nausea (2) Headache (2) Hypomania (2)	
42	Sahraian et al. [63]	30/19	Patients that were suffering from major depression	*C. sativus*, 30 mg/day (capsule)	4 weeks	Headache and nausea (1)		Headache, abdominal discomfort, and nausea (9)	
43	Farokhnia et al. 2014 [64]	68/34	Patients with moderate to severe Alzheimer's disease	*C. sativus* extract, 30 mg/day (capsule)	12 months	Nausea and vomiting (8) Dry mouth (4) Fatigue (4) Dizziness (4) Confusion (1) Sedation (1)		Nausea and vomiting (6) Dry mouth (4) Fatigue (5) Dizziness (6) Confusion (1) Agitation (2) Sedation (3)	
44	Modaghegh et al. [21]	30/20	Healthy volunteers	*C. sativus* extract, 200 and 400 mg/day (tablet)	7 days	Increased their mood (4) Abnormal uterine bleeding (1)			

In these studies, 1479 individuals were assigned to the *C. sativus* groups, and 441 complications were reported, indicating that 29.8% of patients experienced side effects. Among these, approximately 171 cases (38.7%) of the reported side effects were associated with gastrointestinal disturbances, including nausea, vomiting, changes in appetite, and abdominal pain [31, 35]. Simultaneously, 1202 subjects were assigned to the control groups, in which 531 adverse events were recorded, representing 44.1% of patients who encountered adverse reactions (see Table 1). Moreover, Table 2 provides a categorized summary of reported side effects in *C. sativus* intervention groups.

**Table 2:** Categorized summary of reported adverse events in *C. sativus* intervention groups.

Adverse event types	Some specific manifestations	Number of reports	Severity of reported adverse events
Gastrointestinal	Nausea, vomiting, decreased/increased appetite, constipation, abdominal pain, diarrhea, reflux, dry mouth, bloating	171 (38.77%) reports out of 441 total complications	Mild to moderate
Neurological	Fatigue, headache, insomnia, vivid dreams, sedation, dizziness	146 (33.1%) reports out of 441 total complications	Mild to moderate
Psychological	Nervousness, irritability, hypomania, restlessness, anxiety	32 (7.25%) reports out of 441 total complications	Mild to moderate
Cardiovascular	Chest pain, palpitations, tachycardia	Rare	Mild to moderate
Other, sporadic or serious complications	Itching, sweating, cough, joint pain, allergic reaction, bleeding gums, bowel cancer, pancreatitis	Rare and sometimes not considered related to *C. sativus*	Mild to severe

#### 3.1.2 Randomized Trials Reporting No Adverse Events

In total, 35 randomized controlled trials have reported no complications associated with consuming *C. sativus*. A total of 1041 individuals participated in the *C. sativus* groups. Conditions related to metabolic syndrome, such as diabetes and obesity [65, 66, 67], were the most prevalent among patients in these studies, with 7 reports documenting 238 cases. Other study populations included patients suffering from asthma [68], rheumatoid arthritis [69], multiple sclerosis [70], and ulcerative colitis [71], as well as women experiencing post‐menopausal hot flashes [72] or in the late stages of term pregnancy [73]. Additionally, individuals with heart diseases [74], those with schizophrenia [75], anxiety, depression [76], attention‐deficit/hyperactivity disorder [77], and also healthy subjects [78] were also included in the studies. One study examined a single dose of *C. sativus* [79], while the longest intervention period lasted 12 months [80]. More information is available in Table 3.

**Table 3:** Placebo‐controlled trials of saffron preparations reporting no adverse events.

	Trial	*N* total*/N* interventional group	Patient population	Preparation, daily dose	Treatment duration	Adverse events in saffron group
1	Tajaddini et al. [81]	70/35	Patients with type 2 diabetes mellitus	*C. sativus* powder, 100 mg/day (capsule)	8 weeks	“No adverse effect or unwanted symptom was reported in participants.”
2	Kotanidou et al. [65]	81/27	Adolescents with obesity	*C. sativus* stigma powder, 60 mg/day (capsule)	12 weeks	“No adverse events or adverse symptoms were reported by the subjects or their guardians in any study group during the study.”
3	Delam et al. [82]	72/36	Postmenopausal women	*C. sativus* dried stigmas which was boiled once, 30 mg/day	6 weeks	“In the current study, people prescribed 30 mg of saffron daily, which is insignificant, compared to the maximum dose, and it seems that the side effect related to saffron does not threaten people.”
4	Bahrami et al. [70]	80/20	Women with multiple sclerosis	*C. sativus* extract, 15 mg, twice a day (capsule)	12 weeks	No side effects of saffron
5	Roghani et al. [71]	27/18	Patient with ulcerative colitis	*C. sativus*, low‐dose 25 mg, or high‐dose 50 mg, twice daily	8 weeks	“No adverse drug events were reported during the study.”
6	Moghadam et al. [66]	60/15	Obese men with type 2 diabetes mellitus	*C. sativus*, 100 mg/day (pill)	12 weeks	“There were no reports of an adverse event from our saffron or training interventions.”
7	Ahmadikhatir et al. [83]	63/33	Atherosclerosis patients	*C. sativus*, 100 mg/day (capsule)	6 weeks	“The consumption of saffron had no side effects.”
8	Marzabadi et al. [84]	60/30	Mild‐to‐moderate Alzheimer's disease patients	*C. sativus*, 15 mg, twice daily (capsule)	12 weeks	“No adverse events were found in this study.”
9	Tajaddini et al. [67]	70/35	Overweight/obese patients with type 2 diabetes mellitus	*C. sativus*, 100 mg/day (capsule)	8 weeks	“No adverse effects or unwanted Symptoms were reported.”
10	Soheilipur et al. [85]	120/30	Candidates for coronary angiography	*C. sativus* extract, 40 mg, single dose	1 day	“In the current study, participants did not report any side effect for saffron.”
11	Sahebari et al. [69]	55/28	Newly diagnosed rheumatoid arthritis patients	*C. sativus*, 100 mg/day (pill)	90 days	“The patients reported no adverse effect.”
12	Khaksarian et al. [77]	70/35	Children and adolescents with attention‐deficit/hyperactivity disorder	*C. sativus*, 20 or 30 mg/day based on BMI (capsule)(While both groups received 20 or 30 mg/day of methylphenidate)	8 weeks	“No serious side effects were reported during the study.”
13	Lopresti et al. [86]	63/33	Healthy adults with self‐reported poor sleep	*C. sativus* extract (affron), 14 mg, twice daily (tablet)	28 days	“No significant adverse events were reported by participants.”
14	Nemat‐Shahi et al. [87]	164/82	Women with premenstrual syndrome	*C. sativus*, 30 mg/day (capsule)	2 months (within 14 days of the follicular phase)	“The side effects of saffron were not observed during the study.”
15	Zilaee et al. [68]	80/40	Subjects with mild and moderate allergic asthma	*C. sativus* extract, 100 mg/day (capsule)	8 weeks	There were also no serious adverse events
16	Piccardi et al. [80]	31/31 (cross over)	Stargardt disease/fundus flavimaculatus patient	*C. sativus* extract, 20 mg/day (pill)	12 months	The study supplement and placebo were well tolerated and free of any major or minor adverse effect
17	Karimi‐Nazari et al. [88]	75/36	Overweight/obese prediabetic individuals	*C. sativus*, 15 mg/day (pill)	8 weeks	No adverse reactions were reported by the subjects in eithergroup
18	Asadollahi et al. [74]	39/19	Patients with acute ischemic stroke	*C. sativus* extract, 200 mg/day, twice a day (capsule)	3 months	Monitoring of the patients showed that at this dose, crocin had no significant side effects
19	Mirnasrollahi Parsa et al. [89]	76/38	Patients with nonalcoholic fatty liver disease	*C. sativus* extract, 100 mg/day (tablet)	12 weeks	“Nobody expressed any side effects after Saffron supplementation.”
20	Hosseini et al. [90]	80/40	Patients with mild and moderate allergic asthma	*C. sativus*, 100 mg/day (capsule)	8 weeks	“There were no serious adverse events during the study.”
21	Kashani et al. [72]	60/30	Women with postmenopausal hot flashes	*C. sativus* extract, 30 mg/day, 15 mg twice per day (capsule)	6 weeks	“No serious adverse event or death occurred.”
22	Milajerdi et al. [91]	54/27	Patients with type 2 diabetes mellitus	*C. sativus* extract, 30 mg/day (capsule)	8 weeks	“Non‐considerable side effects”
23	Moazen‐Zadeh et al. [92]	76/38	Women with on‐pump coronary artery bypass grafting	*C. sativus* extract, 15 mg/twice daily (capsule)	12 week	“No adverse event was reported that could be associated with saffron or placebo.”
24	Ahmadi et al. [93]	60/30	Women at first childbirth	*C. sativus* stigma powder, 250 mg, every 2 h and up to three dosages (capsule)	With the onset of the active phase of labor	“Saffron seemed to be a good medicine for reducing pain severity and shortening the length of the labor without having complications on mother and fetus.”
25	Kell et al. [94]	128/42	Participants self‐reporting low mood but not diagnosed with depression	*C. sativus* extract, 28 mg/day, 22 mg/day, 28 mg/day (tablet)		“Affron, a natural extract from saffron (*Crocus sativus* L.) improves mood without side effects after 1 month of treatment.”
26	Kianbakht et al. [78]	89/45	Healthy men	*C. sativus* stigma powder, 100 mg/day (tablet)	6 weeks	“Subchronic use of saffron at a dose of 100 mg/day has temporary immunomodulatory activities without hematological, hepatic, renal, or other adverse effects.”
27	Varmazyar et al. [95]	21/7	Active males during a session of eccentric exercise	*C. sativus*, 100 mg/day (capsule)	14 days	“During the use of supplements, there were no side effects due to the use of capsules in the subjects.”
28	Hosseini et al. [96]	13/4	Patients suffering from liver metastasis	*C. sativus*, 50 mg, twice daily (capsule)	3 weeks	“Serious side effect such as anaphylaxis shock was not observed in saffron and placebo groups.”
29	Mohammadzadeh‐Moghadam et al. [97]	50/25	Diabetic men	*C. sativus* gel topical, 28‐g tube (gel)	1 month	“All 50 patients completed the trial without any side effects or complications.”
30	Lashay et al. [98]	30/15	Patients with age‐related macular degeneration	*C. sativus* extract, 30 mg/day (capsule)	6 months	“No major side effects were observed among patients in either the saffron or the placebo group.”
31	Mazidi et al. [76]	60/30	Patients with mild‐to‐moderate mixed anxiety and depression	*C. sativus* extract, 50 mg, twice daily (capsule)	12 weeks	“Side effects were noted during the survey.”
32	Sadi et al. [73]	50/25	Women in the stage of term pregnancy	*C. sativus*, 250 mg, three doses (pill)	24 h	“No adverse event was reported in either of the groups.”
33	Azhari et al. [79]	60/30	Pregnant women	*C. sativus* powder, 250 mg, single dose (capsule)	One dose	“It seems that saffron can be a suitable medicine to reduce labor pain without having any complications for the mother and fetus.”
34	Fadai et al. [75]	44/22	Patients with schizophrenia	*C. sativus* extract, 30 mg/day (capsule)	12 weeks	“In the present study, no side effects were observed.”
35	Nishide et al. [99]	21/10	Healthy adults	*C. sativus* extract, crocin: 0.6 mg/day (tablet)	4 weeks	“No adverse events were observed.”

#### 3.1.3 Randomized Trials Revealing No Adverse Events

In total, 23 studies indicated 639 subjects who did not report any adverse reactions (see Table 4). The diversity of participants in these studies is quite remarkable, as it includes healthy individuals [100], subjects with type 2 diabetes mellitus [101], patients with cognitive disorders [102], and those with atherosclerosis [103], among many other subject populations. The duration of the interventions varied significantly, ranging from a single dose [100] up to a maximum of 12 months [104] (refer to Table 4).

**Table 4:** Placebo‐controlled trials of saffron mono preparations in which nothing was reported about adverse events.

	Trial	*N* total*/N* interventional group	Patient population	Preparation, daily dose	Treatment duration
1	Rajabi et al. [101]	44/11	Obese women with type 2 diabetes	*C. sativus* powdered, 200 mg/day (capsule)	12 weeks
2	Ouerghi et al. [100]	22/22	Healthy active young males	*C. sativus* powdered, 300 mg (capsule)	One dose
3	Gudarzi et al. [105]	40/20	Patients with acute ischemic stroke	*C. sativus* stigmas, 400 mg/day (capsule)	4 days
4	Mojtahedi et al. [106]	48/24	Elderly hypertensive men	*C. sativus*, 200 mg, daily (tablet)	12 weeks
5	Hooshmand‐Moghadam et al. [107]	36/12	Hypertensive men aged 60–70 years	*C. sativus*, 200 mg, daily(tablet)	12 weeks
6	Sakha et al. [104]	43/21	Patients with multiple sclerosis	*C*. sativus, 500 mg, three times a day (pill)	12 months
7	Mobasseri et al. [108]	60/30	Patients with type 2 diabetes mellitus	*C*. sativus powdered, 100 mg/day (capsule)	8 weeks
8	Abassi et al. [109]	20/20	Young active males	*C*. sativus powdered, 300 mg/day (capsule)	Single dose
9	Shahbazian et al. [110]	76/38	Patients with type 2 diabetes mellitus	*C. sativus*, 30 mg/day (pill)	3 months
10	Moravej Aleali et al. [111]	76/38	Patients with type 2 diabetes mellitus Capsule (250 mg)	*C. sativus*, 30 mg/day (capsule)	3 months
11	Ebrahimi et al. [112]	80/40	Type 2 diabetic patients	*C. sativus*, 100 mg/day (tablet)	12 weeks
12	Ebrahimi et al. [113]	80/40	Type 2 diabetic patients	*C. sativus*, 100 mg/day (tablet)	12 weeks
13	Darooneh et al. [114]	60/30	Primiparous women	*C. sativus*, 250 mg/day (capsule)	3 nights
14	Jelodar et al. [115]	40/20	Patients with severe depression	*C. sativus*, 30 mg/day	4 weeks
15	Khatir et al. [103]	63/33	Atherosclerosis patients	*C. sativus*, 100 mg/day	6 weeks
16	Abedimanesh et al. [116]	84/28	Patients with coronary artery disease	*C. sativus* aqueous extract, 30 mg/day (capsule)	8 weeks
17	Kermani et al. [117]	44/22	Subjects with metabolic syndrome	*C. sativus*, 50 mg, twice daily (capsule)	12 weeks
18	Milajerdi et al. [118]	54/27	Type 2 diabetic patients	*C. sativus* extract, 15 mg/day (capsule)	8 weeks
19	Beiranvand et al. [119]	88/44	Female students with premenstrual syndrome	*C. sativus*, 30 mg/day (capsule)	Two menstrual cycles
20	Fukui et al. [120]	57/36	Healthy women volunteers	*C. sativus* odor	20 min
21	Shemshian et al. [121]	105/26	Patients with metabolic syndrome.	*C. sativus*, 100 mg/day (capsule)	12 weeks
22	Tsolaki et al. [102]	35/17	Patients with mild cognitive impairment	not mentioned	12 months
23	Ayatollahi et al. [122]	60/40	Healthy volunteers	*C. sativus*, 200 mg and 400 mg/day (tablet)	7 days (1 tablet/day)

It should be noted that while the overall rate of side effects has been computed, a formal meta‐analysis has not been carried out due to the significant heterogeneity among the included clinical studies. This heterogeneity was noticeable in several areas [1]: the interventions, which differed in the type of preparation (powder vs. extract), duration, and dose [2]; the diversity in studied populations, which varied from healthy volunteers to individuals suffering from chronic diseases; and [3] the method of finding and reporting complications, which was mostly insufficiently described. Therefore, as shown in Tables 1, 3, and 4, a descriptive summary of the data extracted from the clinical trials was considered as an appropriate way to provide a clear picture of the safety profile of this botanical drug in these diverse settings.

### 3.2 Case Reports

In this systematic review, only one case report was included. This case report described a 64‐year‐old male patient with nonvalvular atrial fibrillation who experienced an acute bleeding episode following the intake of rivaroxaban in conjunction with a *C. sativus* supplement. Laboratory tests indicated evidence of platelet dysfunction attributed to *C. sativus*. Discontinuing the use of *C. sativus*, along with rivaroxaban, resolved the bleeding. This study emphasizes the importance of avoiding the combination of direct oral anticoagulants with *C. sativus* due to potential interactions and the associated risk of bleeding [123].

### 3.3 Methodological Quality Assessment of the Studies

**Figure 2:**
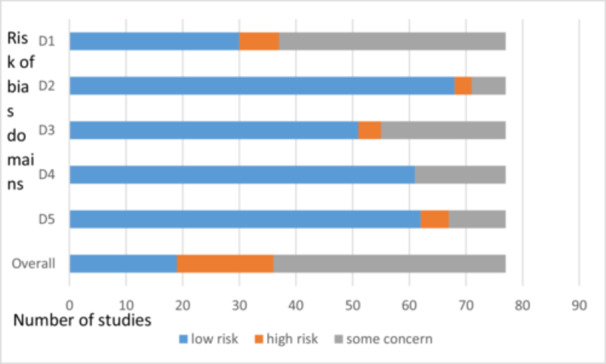
Bias assessment in clinical trials on saffron preparations that reported on adverse effects, whether present or absent, using Cochrane's Risk of Bias‐2 (ROB‐2) tool. D1: Randomization process. D2: Deviations from the intervention. D3: Missing outcome data. D4: Measurement of the outcome. D5: Selection of reported results.

**Figure 3:**
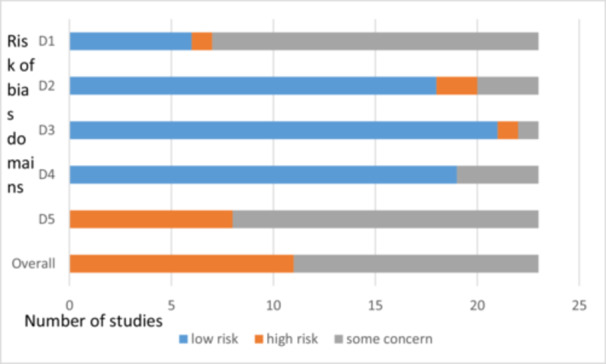
Bias assessment in clinical trials on saffron preparations that did not mention adverse events using Cochrane's Risk of Bias‐2 (ROB‐2) tool. D1: Randomization process. D2: Deviations from the intervention. D3: Missing outcome data. D4: Measurement of the outcome. D5: Selection of reported results.

Based on the reported content of studies on adverse events, the trials were grouped into two categories, and risk of bias assessment graphs were presented for each category independently: (1) Bias assessment in clinical trials on saffron preparations that reported on adverse effects, whether present or absent, using Cochrane's ROB‐2 tool (Figures 2) and (2) Bias assessment in clinical trials on saffron preparations that did not mention adverse events using Cochrane's ROB‐2 tool (Figure 3).

## 4 Discussion

*C. sativus* is known all over the world for its ability to help manage cardiovascular disorders, immunologic disorders, cancers, metabolic syndrome, gynecological, and neurodegenerative diseases [124, 125]. There are numerous reviews regarding the supplementation of *C. sativus* in human studies [4, 126]. Still, there has been no systematic review that has investigated the adverse effects. One of the most crucial issues that needs to be addressed in herbal medicines is the glaring lack of safety data. Furthermore, it is often the case that people misguidedly think about the safety of these medicines as remedies because they are “natural” [127]. There is evidence that suggests that the adverse events associated with herbal medications are not often reported when compared to the adverse reactions from allopathic medicines [128].

The current systematic review of the safety profile of *C. sativus* presented in the tables provides a comprehensive overview of adverse events associated with *C. sativus* supplementation, based on all clinical trials and a case report.

The distribution of clinical studies on *C. sativus* by field shows that the largest volume of research (54 studies) has focused on the “psychiatry and neuropsychiatry” field, reflecting the significant research interest in the role of *C. sativus* in mood, anxiety, and cognitive disorders. In the next ranks, the fields of “metabolic” with 21 studies and “women and reproductive health” with 16 studies are in the order of precedence, indicating the extensive applications of this plant in the management of diabetes, metabolic syndrome, as well as disorders related to the menstrual cycle, menopause, and pregnancy. In contrast, fields such as “ophthalmology, rheumatology, gastroenterology, and respiratory”, with fewer than five studies each, suffer from a significant research gap, requiring the design of larger and more standardized studies in the future to strengthen the evidence in these fields. Also, the dispersion of adverse event reports across different schedules may be related not only to the nature of the intervention itself but also to differences in monitoring methods, sensitivity of the study populations, and quality of reporting.

It is worth noting that almost a quarter of the trials reviewed (23 out of 102) did not report any adverse events. This lack of reporting does not mean that there was no harm, but rather that safety outcomes in these studies were not systematically checked or recorded. As highlighted in the methodological guide for systematic reviews of adverse effects, such insufficient reporting can express notable bias, causing an underestimation of the extent of the risk. Trials that do not describe harm‐monitoring methods in detail may be of lower quality in terms of safety outcomes, as accurate assessment of adverse events needs prospective and active observations [129, 130]. In the present review, the calculated overall adverse event rate (about 17.5%) and description of the safety profile of *C. sativus* are drawn only from those trials that actively collected data regarding safety. Although most of the reported adverse events across the included trials were mild and self‐limiting, the probability of underreported events cannot be ruled out. Future trials should follow standard guidelines for monitoring and documenting adverse events to enhance the comparability and reliability of safety data.

Roughly, 78% (44 out of 102) of the trials focusing on mono‐preparation of *C. sativus* claimed to have addressed safety and complications monitoring and reporting (Tables 1 and 3). In this group of studies, 56% (44 out of 79) claimed that they have documented one or multiple adverse events during the intervention with *C. sativus*. In total, 2520 participants across these studies were given *C. sativus* with different doses, dosages, and duration, which in turn led to claiming 441 adverse event cases, the most of which being slight and self‐limiting. Hence, the approximate rate of adverse reactions due to the use of *C. sativus* is about 17.5%.

As indicated in Table 1, the majority of the adverse effects reported were nonserious, with gastrointestinal complications ranking first [31, 33, 35]. Gastrointestinal side effects were mild to moderate, such as nausea, abdominal pain, and changes in appetite. Future study designs of standardized crocin (as the most important bioactive compounds in *C. sativus*) concentrations and different dosing patterns (e.g., single dose vs*.* divided doses) could help clarify the dose–response relationship and reduce these side effects. Reports of vivid dreams or sleep changes were mainly observed in studies of anxiety disorders or depression. This may indicate a potential neurochemical interaction between *C. sativus* compounds (with possible effects on the GABA or serotonin system [131]) and neurotransmitters involved in these disorders. In other words, psychiatric populations may be more sensitive to some of the neuromodulatory effects of saffron due to their specific neurobiological background. This hypothesis requires further investigation in mechanistic studies.

However, the findings of this systematic review and a single case report that were associated with the concomitant use of *C. sativus* with rivaroxaban with an acute bleeding episode highlight the need to know the potential interactions of *C. sativus* with anticoagulants [123]. Previously, the potential antiaggregatory properties of *C. sativus* have been investigated in human platelets. *C. sativus* seemed to influence the regulation of calcium influx in activated platelets. This herb dose‐dependently reduced platelet aggregation caused by epinephrine, ADP, and collagen. Moreover, studies using computational methods have shown a common binding site for *C*. *sativus* carotenoids and aspirin in cyclooxygenase I enzyme [123, 132, 133]. Therefore, it seems essential for clinicians, especially when prescribing *C. sativus* to patients treated with anticoagulants or antiplatelet drugs, to be aware of these potential mechanisms and to closely monitor bleeding symptoms and coagulation times. It is recommended that patients should be educated about the warning signs of bleeding and be clinically monitored.

From our analysis shown in Table 1, which lists the frequency of adverse events in trials evaluating the effects of *C. sativus*, there are some striking observations. The patients in the study group who consumed *C. sativus* seem to have a lower proportion of adverse events as compared to patients in the control group. The interpretation of this finding should be done with caution and may be related to different factors: (1) Heterogeneity in monitoring and reporting: A considerable defect in the methods of many included studies is the passive approach to collecting adverse events data. Less strict monitoring or that relying more on spontaneous reporting in the *C. sativus* group may lead to underreporting the mild or moderate adverse events. On the other hand, control groups, particularly those getting an active comparator such as anxiolytic agents, might have undergone more structured monitoring for known complications. This could lead to higher detection rates of adverse events. This situation reflects the known difficulties in assessing harm, in which reporting bias is common. (2) Nocebo effect: Nocebo effect occurs when individuals in the control group, especially those getting an inert placebo, report vague symptoms because of negative expectations [134]. This effect is significant in clinical trials on anxiety or depression, which are commonly studied in *C. sativus* research. (3) Expected side effects of control interventions: Active drugs with known side effects, such as SSRIs, may be used as a control in trials. The higher incidence of complications in these groups reflects their known toxicity, not the absence of complications with *C. sativus*.

According to the findings of this systematic review, it seems that certain populations, such as patients with psychiatric disorders and the elderly, may be more susceptible to adverse events associated with *C. sativus* consumption. In studies involving older participants (especially those over 50 years of age), serious adverse events (such as colon cancer) were observed [27, 42].

Responsible interpretation of the findings of this review requires careful distinction between reported adverse events and a causal relationship with *C. sativus*. For example, the occurrence of serious adverse events such as bowel cancer in trials of age‐related macular degeneration [27] warrants special attention. A close examination of these studies revealed that:

1. Participants were predominantly elderly people (> 50 years) with a high baseline risk for chronic diseases and age‐related complications.

2. The principal investigators of these trials themselves emphasized that these serious events were unlikely to be related to *C. sativus* supplementation.

Although these adverse events were not necessarily attributed to *C. sativus* consumption, older age is known to be an independent risk factor for adverse events. Therefore, it is suggested that targeted safety monitoring in these vulnerable subgroups should be considered in therapeutic applications of *C. sativus*, especially at high doses or long‐term use. This could include regular monitoring of blood markers, liver and kidney function, careful assessment of neuropsychiatric complications, and careful recording of any gastrointestinal or cardiovascular complications.

We did not find a clear pattern that stigma powder or aqueous or hydroalcoholic extracts caused more side effects. Most studies did not report sufficiently about the precise standardization of these products (e.g., the exact amount of crocin or safranal). This lack of standardization information makes it difficult to make accurate comparisons between the types of products. At common doses (15–100 mg/day), increasing the dose did not result in a clear and consistent increase in side effects. Within this dose range, most side effects were mild and transient. However, in the few studies that used higher doses (e.g., 400 mg/day), although no major side effects were reported, the small sample size of these studies precluded definitive conclusions. Regarding the duration of use, there was no clear pattern of accumulation of side effects with long‐term use (up to 12 months) in the available data.

Finally, to guide safe clinical use, the following points should be considered:

1. Distinction between oral and therapeutic doses: The findings of this review are mainly related to medicinal/therapeutic doses (usually 15–100 mg/day and in some cases up to 400 mg). Consumption of *C. sativus* as a spice in conventional dietary amounts (usually below 30 mg/day) is considered safe based on the available evidence and long history of use.

2. Identification of high‐risk populations: Special caution should be exercised when prescribing or consuming *C. sativus* (especially at therapeutic doses) in the following groups:

– The elderly: due to pharmacokinetic alterations, comorbidities, and polypharmacy.
– Patients treated with anticoagulant or antiplatelet drugs: due to the risk of interference and increased bleeding (as confirmed in a case report).
– Patients with underlying psychiatric disorders: due to potential increased susceptibility to neuropsychiatric complications.
– Pregnant and lactating women: due to limited safety data in this group.

3. Standardization of dosage and clear labeling: The wide heterogeneity in the product type (stigma powder vs. extract), standardization method, and reporting of active compounds (crocin, safranal, picrocrocin) is the main obstacle to comparability of studies and accurate determination of dose‐response relationships. It is recommended that all *C. sativus* products (especially commercial supplements) should have clear labeling that states the type of plant part, extraction method, and amount of key compounds.

4. Postmarket surveillance for commercial products: Given the increasing popularity of standardized *C. sativus* supplements, it is essential to establish active pharmacovigilance and adverse event reporting systems for these products to detect rare or delayed adverse events that may not be identified in short‐term trials.

In conclusion, while *C. sativus* is generally well tolerated at conventional doses and for short periods, its therapeutic use requires a cautious approach, awareness of drug interactions, identification of high‐risk patients, and support for future research to standardize and monitor long‐term safety. This balanced framework will help physicians and consumers to benefit from the potential benefits of *C. sativus* in a safe and informed manner.

## 5 Conclusion

*C. sativus* is generally well tolerated at usual doses and for short periods, and its side effects are often mild and transient. However, this general conclusion should be accompanied by important practical considerations. Patients with underlying psychiatric disorders, the elderly, and especially those taking concomitant anticoagulant agents, appear to be at higher risk for potential side effects and require increased caution and monitoring. Regarding dosage, although no clear increase in side effects has been observed within the common range (15–100 mg/day), the use of much higher or prolonged doses outside this range should be accompanied by close clinical monitoring, and a definitive dose threshold for side effects cannot currently be determined.

From a clinical perspective, it is essential that physicians carefully review the patient's complete drug history before prescribing *C. sativus*, especially about concomitant anticoagulants. It is also important to inform patients about warning signs such as unusual bleeding or mood changes. Overall, although *C. sativus* appears to be safe in food and supplement applications at conventional doses, its therapeutic use at high doses or for long periods requires a cautious approach accompanied by medical supervision, and future studies should provide a more complete picture of its safety profile, focusing on sensitive groups and drug interactions.

## 6 Limitations, Gaps, and Priorities in Future Research

The use of clinical trials involving *C. sativus* has a rich potential for research enhancement. However, it is essential to pay attention to the concerns mentioned below to better design future studies.

A considerable methodological limitation present in many of the included studies was the inadequate reports regarding the type of *C. sativus* extract (aqueous, hydroalcoholic) that was applied in preparations. Moreover, important standardization parameters, such as the key active constituents (e.g., picrocrocin, crocin, safranal), were sometimes unreported. The lack of such detailed information offers a major restriction for the explanation of safety data. The pharmacokinetic profile, bioavailability, efficacy, and even safety of *C. sativus* may differ markedly based on the method of extraction and its active constituent concentrations. The observed difference in side‐effect rates across trials may be partly related to these unmeasured variances in the product composition. To improve the reproducibility and reliability of future studies, we strongly suggest that studies on *C. sativus* should be reported in a standardized manner. This reporting should incorporate the details of the plant part used (e.g. petal, stigma), the method of the extraction and solvent used, the dosage form, as well as quantitative levels of key bioactive compounds or the chromatographic fingerprint. Such methods would allow for further comparisons between trials, facilitate dose–response evaluations, and contribute more accurate insights into causal relationships between certain *C. sativus* products and their adverse events.

While it is important to point out that the approximate incidence rate of side effects resulting from *C. sativus* is about 17.5%, this review shows the gaps that emphasize the need for further safety evaluations. Due to the considerable heterogeneity in the dose, dosage form (extract vs. powder), and duration of *C. sativus* use in the included studies, it was not possible to perform a quantitative dose–response analysis in this systematic review. Most of the reported adverse events were mild at common doses (mainly below 100 mg/day). However, prospective studies with uniform design and active monitoring of adverse events are necessary to more accurately determine the relationship between the dose and adverse events, especially at high doses or long‐term use.

A significant proportion of reported adverse events (such as mild gastrointestinal symptoms, headache, or sleep changes) are subjective in nature. In trials in which the control group received an inactive placebo, there is a possibility of a nocebo effect—that is, reporting adverse events due to negative expectations. This could increase the baseline adverse event rate in both groups and make it difficult to accurately assess the exact contribution of *C. sativus*.

As noted earlier, adverse event monitoring and reporting methods were highly heterogeneous across studies and often passive (based on spontaneous reporting). This heterogeneity makes direct comparisons of adverse event rates between studies challenging and increases the likelihood of underreporting, especially for mild adverse events.

Many of the included trials had relatively small sample sizes. Small studies are often underpowered to detect rare but potentially serious adverse events, which could underestimate the true risks. Several trials have used standardized commercial *C. sativus* products, which requires consideration of potential conflicts of interest. Also, the generalizability of the findings of these studies to other forms or brands of *C. sativus* should be considered with caution.

Longer‐term trials (e.g., 12‐month trials) have a greater chance of observing rare or delayed‐onset adverse events. In contrast, short‐term studies tend to identify more common and early adverse events. This emphasizes the need for longer‐term studies with active safety monitoring, especially for evaluating chronic saffron use. Postmarketing adverse event reporting systems can also play a critical role in completing this picture.

It is also important to consider potential drug interactions (e.g., anticoagulants) at different dose levels of *C. sativus* usage. More studies are warranted on the *C. sativus* pharmacokinetics and its active ingredients, to clarify the causative mechanisms that give rise to the side effects.

## Author Contributions

Conceptualization: Fatemeh Sadat Hasheminasab and Seyede Maryam Najibi. Methodology: Fatemeh Sadat Hasheminasab. Validation: Seyede Maryam Najibi, Mohammad Hashem Hashempur, and Mahdie Hajimonfarednejad. Formal analysis: Fatemeh Sadat Hasheminasab. Investigation: Seyede Maryam Najibi. Data curation: Mahdie Hajimonfarednejad and Seyede Maryam Najibi. Writing – original draft: Fatemeh Sadat Hasheminasab. Writing – review and editing: Mohammad Hashem Hashempur, Fatemeh Sadat Hasheminasab, and Seyede Maryam Najibi. Supervision: Fatemeh Sadat Hasheminasab and Mohammad Hashem Hashempur. All authors have read and approved the published and final version of the manuscript.

## Funding

The authors have nothing to report.

## Conflicts of Interest

The authors declare no conflicts of interest.

## Transparency Statement

The lead authors Mahdie Hajimonfarednejad and Seyede Maryam Najibi affirm that this manuscript is an honest, accurate, and transparent account of the study being reported; that no important aspects of the study have been omitted; and that any discrepancies from the study as planned (and, if relevant, registered) have been explained.

## Data Availability

All data are available upon request to the corresponding authors. S.M.N. and M.H. had full access to all of the data in this study and take complete responsibility for the integrity of the data and the accuracy of the data analysis.
